# Nature curing cancer – review on structural modification studies with natural active compounds having anti-tumor efficiency

**DOI:** 10.1016/j.btre.2015.01.005

**Published:** 2015-01-24

**Authors:** Gurpreet Kaur, Neelam Verma

**Affiliations:** Department of Biotechnology, Punjabi University, Patiala 147002, Punjab, India

**Keywords:** Cancer, Structure–activity relationship studies, Natural anti-cancer agents, Cytotoxicity, Anti-proliferating activity

## Abstract

Cancer treatment has raised many drugs and radiation therapies whose side-effects are visible. Ongoing research throughout the world for effective treatment mainly concentrates on methods either in the form of drugs or therapies against this lethal disease. However returning to ayurvedic roots enlightens the fact that nature has many efficient components with anti-cancerous, anti-proliferating and anti-angiogenesis effects. Evidences confirm the participation of plants extracts in synthesizing many medicines against already existing and even emerging diseases. Structure activity relationship (SAR) studies and structural modifications are helping in observing the basis of compounds characteristics to exhibit inhibitor’s nature against carcinogenic agents by modifying parent compounds for creating an improved and potent compound. Many components are under clinical trials but most of them still need attention. In this review an attempt has been made to focus on the natural components gifted by nature and even included in our diet with their structures and sources that could be supportive in designing drug either by computational methods or by experimental methods.

## 1 Introduction

Most primitive and effective method from pre-historic time to heal injuries, cure disease and relieve suffering is treatment from plants extract. Every part of plant from root to tip participates in the production of drugs against many diseases. According to World Health Organization most lethal and leading cause of death worldwide accounting for 7.6 million deaths (13% of all deaths) in 2008 is Cancer. Researchers use natural components, synthetic compounds and even micro-organisms for making an active drug with the capability to distinguish between healthy cells and tumor cells. Though many natural compounds are under clinical trials yet compounds with anti-cancerous activities still not under clinical trial cannot be neglected

Tian Xian, sophora root, zedoania, rhubarb root and rhizome, mistletoe, cleavers, sweet violet, hot pepper, capsaicin, neem, country gooseberry, selaginella are some herbs still under investigation for their active ingredient acting as anti-tumor agents as well as for SAR studies.

## 2 Turmeric

For centuries in Indian subcontinent spice named Turmeric is readily used as food ingredient as well as an ayurvedic medicine [1]. The major anti-cancer compound in turmeric is curcumin ((1E,6E)-1,7-bis(4-hydroxy-3-methoxyphenyl)-1,6-heptadiene-3,5-dione) which has been able to suppress many biological factors responsible for the proliferation of cancer cells, for instance NF-kB, IkBα kinase, by induction in apoptosis pathway through activation of caspase-8, BID cleavage and cytochrome C release in human lung, breast, prostate tumor cell lines and many myeloma cells [2], [3], [4], [5], [6], [7], [8]. Further curcumin administration exerted a gradual deterioration in tumor growth rate and gelatinolytic activities of matrix metaloproteinase-9 with a significant increase in animal survival time. Moreover suppression of hemoglobin concentration in tumor studies gave a clear indication of angiogenesis process inhibition. Overall results conclude curcumin as the potent inhibitor for tumor cells [9]. But due to poor absorption of curcumin in bloodstream certain derivatives have been proposed which may help in enhancing its bioavailability. Xu et al. in their studies during 2012, focus on structure–activity analysis between androgen receptor and curcumin derivatives. They suggested that for getting improved cytotoxic activity modifications favors the presence of H-bond acceptors (negatively charged) at R1, R2, R3 and R4 position, hydrophobic substituents attached to linker, biphenyl rings at both the sides and bulky groups at C-4 position of linker (Table 1) [10].

**Table 1:** Anti-cancer compounds with their source, structures and location.

Anti-cancerous agent	Active compound		Location
	Name	Structure	
Turmeric (*Curcuma longa*)	Curcumin ((1 E,6E)-1,7-bis(4-hydroxy-3-methoxyphenyl)-1,6-heptadiene-3,5-dione)		South and Southeast Asia
Ashwagandha (*Withania somnifera*)	Withaferin-A ((4β,5β,6β,22 R)-4,27-dihydroxy-5,6:22,26-diepoxyergosta-2,24-diene-1,26-dione)		India, Pakistan, Afghanistan, Spain, parts of the Middle East, Africa and the cancry Islands
Honey	Caffeic acid phenethyl ester (CAPE) (2-phenylethyl(2E)-3-(3,4-dihydroxyphenyl)acrylate)		Worldwide
Garlic (*Allium sativum*)	Diallysulfide (4,5-dithia-1,7-octadiene) or allylmethylsulfide (3-(methylsulfanyl)-1-propene)		Native to central Asia, staple in the Mediterranean region, frequent seasoning in Africa, and Europe
Fruits	Ellagic acid (2,3,7,8-tetrahydroxychromeno[5,4,3-cde]chromene-5,10-dione) (newly discovered in red berries and pomegranate)		Worldwide
Red grape (*Vitis vinifera*)	Resveratrol (3,5,4′-trihydroxy-trans-stilbene)		Native to the Mediterranean and Central Asia
Tomato (*Solanum lycopersicum*), water-melon (*Citrullus lanatus*), guava (*Psidium*)	Lycopene (ψ,ψ-carotene)		Native to South America and was spread worldwide
Saffron (*Crocus sativus*)	Crocetin ((2E,4E,6E,8E,10E,12E,14E)-2,6,11,15-tetramethyl-2,4,6,8,10,12,14-hexadecaheptaenedioic acid)		Native to Southwest Asia was later brought to parts of North Africa, North America, and Oceania
Butterfly ginger (*Hedychium coronarium*)	Alpha terpineol 9 2-(4-methyl-3-cyclohexen-1-yl)-2-propanol) and [6]-gingerol ((5S)-5-hydroxy-1-(4-hydroxy-3-methoxyphenyl)-3-decanone) poly-phenolic constituents gingerols and zerumbone ((2E,6E,10E)-2,6,9,9-tetramethyl-2,6,10-cycloundecatrien-1-one)		Himalayas region of Nepal and India
Green Tea (*Camellia sinensis*)	EGCG OR EGC [epigallocate-chin gallate] ((2S,3S)-5,7-dihydroxy-2-(3,4,5-trihydroxyphenyl)-3,4-dihydro-2H-chromen-3-yl 3,4,5-trihydroxybenzoate)		Native to China and other countries in the Orient, Far East and India
Grape Seeds (*Vitis vinifera*)	Gallic acid (3,4,5-trihydroxybenzoic acid)		From Western Europe to the Persian shores of the Caspian Sea
Apricot kernels (*Prunus armeniaca*)	Laetrile		Europe, South Australia, central Asia and around the Mediterranean
Corn (*Zea mays*)	Ferulic acid ((2E)-3-(4-hydroxy-3-methoxyphenyl) acrylic acid)		Native to the Balsas River valley in south-eastern Mexico, Canada and the United States, Southern Africa
Vegetables	Sulforaphane (1-isothiocyanato-4-(methylsulfinyl) butane)		Worldwide
Flex seeds (*Linum usitatissimum*)	Lignans and an omega-3 fat called alphalinolenic acid		Native to the region extending from the eastern Mediterranean to India
Beet (*Beta vulgaris*)	Betacyanin		Throughout the Mediterranean, the Atlantic coast of Europe, the Near East, and India
Hop (*Humulus* *lupulus*)	Chalcones including xanthohumol		Germany, UK, USA, Poland, China
Cats claw (*Uncaria* *tomentosa*)	Quinic acid ((1S,3R,4S,5R)-1,3,4,5-tetrahydroxycyclohexanecarboxylic acid)		Found in the tropical jungles of South and Central America
Maitake mushrooms (*Grifola frondosa*)	Irofulven ((6′R)-6′-hydroxy-3′-(hydroxymethyl)-2′,4′,6′-trimethylspiro[cyclopropane-1,5′-inden]-7′(6′H)-one)		Native to the northeastern part of Japan and North America
Spirulina (*Arthrospira*)	C-phycocyanin		Worldwide
Red clover (*Trifolium* *pratense*)	Genistein (5,7-dihydroxy-3-(4-hydroxyphenyl)-4H-chromen-4-one) [prevent angiogenesis]		Native to Europe, Western Asia and northwest Africa, but planted and naturalised in many other regions
Tripterygium (*Tripterygium* *wilfordii*)	Celastrol ((2R,4aS,6aS,12bR,14aS,14bR)-10-hydroxy-2,4a,6a,9,12b,14a-hexamethyl-11-oxo-1,2,3,4,4a,5,6,6a,11,12b,13,14,14a,14b-tetradecahydro-2-picenecarboxylic acid), triterpene		China
Marine natural product	Lamellarins		Marine region
Shark liver oil	Alkylglycerols		Ocean
Aloe vera	Aloe-emodin (1,8-dihydroxy-3-(hydroxymethyl)-9,10-anthraquinone)		Worldwide
Saw plametto (*Serenoa* *repens*)	Beta-sitosterol ((3β)-stigmast-5-en-3-ol), stigmasterol ((3β,22 E)-stigmasta-5,22-dien-3-ol)		Endemic to the southeastern United States, along the Atlantic and Gulf Coastal plains, southern Arkansas, Florida
Radium weed/ petty spurge/ cancer weed/ milk weed (*Euphorbia* *peplus*)	Ingenol mebutate ((1S,4S,5R,6R,9S,10R,12R,14R)-4,6-dihydroxy-7-(hydroxymethyl)-3,11,11,14-tetramethyl-15-oxotetracyclo[75,101,5010,12]pentadeca-2,7-dien-5-yl (2 Z)-2-methyl-2-butenoate)		Australia, New Zealand, North America, and other countries in temperate and sub-tropical regions
Pau D’Arco (*Tabebuia*)	Lapachol (2-hydroxy-3-(3-methyl-2-buten-1-yl)-1,4-naphthoquinone)		From northern Mexico and southern Florida south to northern Argentina, Caribbean islands of Puerto Rico, Hispaniola (Dominican Republic, Haiti), Jamaica, Trinidad and Tobago and Cuba, Brazil
Graviola (*Annona muricata*)	Acetogenins		Native to Mexico, Cuba, Central America, the Caribbean and northern South America: Colombia, Brazil, Peru, and Venezuela, sub-Saharan African countries, some areas of Southeast Asia as well as in some Pacific islands
Chuchuhuasi (*Maytenus* spp.)	Maytenin, maytansine ((4β,11E,13E)-maytansine)		Worldwide, commonly found in South America
Wormwood (*Artemisia* *absinthium*)	Artemisinin ((4S,5R,8S,9R,12S,13R)-1,5,9-trimethyl-11,14,15,16-tetraoxatetracyclo[103,104,1308,13]hexadecan-10-one)		Native to temperate regions of Eurasia and northern Africa
Mushrooms (*Agaricus* *bisporus*)	Active hexose correlated compound (acetylated α-glucan, β-glucan)		Worldwide
Coffee	Chlorogenic acid ((1S,3R,4R,5R)-3-{[(2E)-3-(3,4-dihydroxyphenyl)-2-propenoyl]oxy}-1,4,5-trihydroxycyclohexanecarboxylic acid)		Latin America, Southeast Asia, South Asia and Africa
Oldenlandia (*Oldenlandia diffusa*)	Oleanolic acid its isomer ursolic acid, both pentacyclic triterpenes		China

## 3 Ashwagandha

Ashwagandha has powerful antioxidant properties to seek as well as to destroy free radical and simultaneously boost the activity of antioxidant enzymes (for example superoxide dismutase and catalase) in order to prevent adverse effect on nervous system. Moreover in-vitro studies to treat many types of cancer cell lines such as human breast, lung and colon cancer against common cancer chemotherapy drugs gave comparable results thus proving it as an anti-tumor agent. The compound active for anti-cancerous property in this plant is withaferin A ((4β,5β,6β,22R)-4,27-dihydroxy-5,6:22,26-diepoxyergosta-2,24-diene-1,26-dione) [11], [12], [13], [14]. Its property to inhibit angiogenesis and furthermore to act as adjunct to chemotherapy treatment by protecting the gradual deterioration in white blood cells called neutrophils without any contrast, enlighten its performance as key step in fighting cancer [15], [16], [17], [18]. Animal toxicity studies designate this astonishing plant safe and well tolerated [19]. In-vitro studies of withaferin and its derivatives on P388 cells performed by Fuska et al. predict through SAR studies that dissociation of double bond at C2=C3 position shows notable decline in cytotoxicity in all derivatives. In addition carbonyl group at C4 position results in enhancement of effect of agent while no change in biological functions can be seen on dissociation of double bond at C24=C25 or on removal of OH group from C27 (Table 1) [20]. Further Zhang et al. in search of bioactive compounds isolated 29 withanolides and prepared derivatives from them followed by anti-proliferation testing on array of cell lines. Out of all 15 withanolides showed better anti-proliferactive activity against various cell lines. From their own research and the data obtained from literature it was concluded that 5β,6β-epoxy group or 5α-chloro-6β-hydroxy function in ring B, conjugated ♠^2^-1-oxo function in ring A and esterification of hydroxyl group enhances the cytotoxic effect of compound while presence of —OH and —OR groups at C-4,7,11,12,14,15,16,17,18,19,20,23,24 and 27 does not have significant role in improving the cytotoxic effect of compound. They also suggest glycosylation of withanolide as a reason decreased anti-proliferative action [21].

## 4 Honey

The book ‘Honey Revolution’ written by Dr. Ron Fessenden states that minute traces of floral bioflavonoids generally known as antioxidants contribute majorly by raising antioxidant levels within the cells [22]. Testing of honey as anti-cancerous agent is yet in practice. Researchers found caffeic acid phenethyl ester (CAPE) as active compound in bee propolis proficient to inhibit early-stages of prostate cancer. Its anti-mitogenic and anti-carcinogenic characteristics were capable to inhibit NF-kB pathways in cancer cells. Experiments on tumor growth of LNCaP xenografts in mice significantly illustrated the results [23]. In addition, preventive and curative effect of bee products on tumor cells approves its anti-cancerous nature. SAR studies on CAPE suggest the significance of number of hydroxyl groups. The fact was observed by replacing and adding hydroxyl groups in CAPE structure. Interchange in position of hydroxyl group in CAPE from 3,4-dihydroxy to 2,5-dihydroxy resulted in enhancement of potency while replacement of hydroxyl group with methyl ethers and addition of third hydroxyl group resulted in loss of inhibiting effect of compound. At the same point an increase in alkyl chain proved to be an insignificant compound related to inhibitory effect (Table 1) [24]. In addition to CAPE other polyphenols believed to be effective against cancer disease are caffeic acid (CA), chrysin, galangin, quercentin, kaempferol, acacetin, pinaocembrin, pinobanksin and apigenin. Orsolic et al. in 2005 concluded the significant suppression activity of CA and CAPE on human HeLa cervical tumor cell line through in-vitro experiments [25].

Chrysin (5,7-dihydroxy-2-phenyl-4H-chromen-4-one) is another anti-cancer active compound in honey. Unfortunately its anti-cancer characteristics have rarely been studied. Structural studies showed that chrysin has similar structure to flavone structure with a difference of presence of two hydroxyl groups at position 5 and 7 at ring A. These two hydroxyl groups with 2, 3 double bond at ring C and attachment of B and C rings at position 2 (Table 1) are considered as the effective regions for apoptotic properties in certain human cancer cell lines like cervical cancer, leukemia, esophageal squamous carcinoma, malignant glioma, breast carcinoma, prostate cancer, non-small cell lung cancer and colon cancer while in many cases lower potency rate have been noted. Many in-vitro experiments demonstrated that instead of using only chrysin, either modification of chrysin or combination of chrysin with other flavonoids could improve the effectiveness of apoptotic factor [26]. In accordance to Monasterio et al. the presence of at least two hydroxyl groups at position 3, 5 or 7 in form of 3–5, 5–7 or 3–7, were needed for proper apoptotic effect. The possibility was based on the comparison between structural similarity of chrysin, galangin and various similar compounds which differ only in positioning of hydroxyl group. The conclusion was formed after analyzing the better apoptotic activity of 3,7-dihydroxyflavone, 5,7-dihydroxyflavone (chrysin), 3,5,7-trihydroxyflavone (galangin) when compared with other compounds i.e., 5-hydroxyflavone, 7-hydroxyflavone, 4′,7-dihydroxyflavone, and 7,8-naphtoflavone [27].

## 5 Garlic

With the starting of 1957, researchers were involved to determine the facts in favor of garlic for treatment of cancer. Capability of garlic extract to bring irregularities and to scatter the chromosomes in the cancer cells introduced a new compound for treatment. Later, reversed tumor development was not only seen in mice but also in mammary tumors and sarcomas. Population consuming garlic and cancer proliferation gave an inverse trend of overpowering cancer development [28]. Most noteworthy evidences aroused from the study performed on digestive tract tumors, for instance – gastric cancer, stomach cancer, esophageal cancer and colorectal cancer [29]. Effectiveness of the extract could be seen in not only fresh-garlic extract but in aged-garlic extract as well [30]. Allium active constitutes namely allysulfides such as diallyldisulfide or allylmethyltrisulphide possibly relates to inhibit carcinogenesis by stimulating Phase II conjugation systems and simultaneously enhancing glutathione S-transferase (GST) which help in detoxifying carcinogen free-radicals. Moreover it assists the synthesis of DNA regulation [31]. Studies reveal allyl group of this compound as factor responsible for suppressing premalignant lesions caused by azoxymethane. Inhibition in carcinogenic activity could be seen when single allyl-chain is linked to sulfur atom (Table 1). The compound was also able to inhibit cytochrome p4502E1 (CYP2E1) isoform which is usually believed to activate wide range of low molecular weight compounds [32]. In 2010, Kaschula et al. worked on garlic derived organosulfur compounds and suggested that lipid soluble anti-cancer agent in crushed garlic, namely ajoene, diallyl sulfide, diallyl disulfide, diallyl trisulfide are more potent as compared to water soluble S-allylmercaptocysteine and S-allylcysteine. They also highlighted common structural feature in ajoene and garlic-derived allyl sulfide important factor responsible for anti-tumor activity is allyl sulfide or polysulfide backbone. It was hypothesized that performance of this group lies in vinyl disulfide core. Remarkably, in ajoene replacement of allyl end group (sulfoxide end) with an alkyl or benzyl group generated its analogs with improved or similar activity [33].

## 6 Fruits and vegetables

Ellagic acid being a natural phenolic antioxidant is consumed through many fruits and vegetables. Inhibition of anti-proliferative activity and induction of apoptosis involves the activation of caspase cascade [34]. It is a planar molecule with skill to inhibit CYP1A1-dependent activation of benzo[a]pyrene, DNA binding of certain carcinogens and control various anticarcinogenic activities. Ellagic acid has the property to inhibit CYPlAl-dependent activation of benzo[a]pyrene thus in turn inhibiting polycyclic aromatic hydrocarbon-induced tumorigenesis. Barch et al. discussed the importance of hydroxyl, lactone groups and 3-hydroxyl and 4-hydroxyl group. They concluded that 4-hydroxyl group is needed to inhibit CYP1A1-dependent activation of benzo[a]pyrene activation. Moreover in case of directly inhibiting benzo[a]pyrene diolepoxide both 3-hydroxyl and 4-hydroxyl groups were essential. The facts were verified through study with ellagic acid and its chemical analogs. (Table 1) [35].

## 7 Tomato

Lycopene is the carotene found in tomato. Consumption of tomato highly drops the risk factors associated to reactive oxygen species (ROS) mediated diseases. It is suggested that lycopene in combination with other phytonutrients can gradually increase the consequence to inhibit several pathways related to cancer cells growth as well as interfere in mitogenic pathway of IGF-I [36], [37]. Lycopene property to lack any electrophilic group gave similar results in activation of electrophile/antioxidant response element (EpRE/ARE) transcription system and in inhibiting breast and prostate cancer cell growth as well. Linnewiel et al. used a series of characterized mono as well as diapocarotenoids for displaying results for EpRE/ARE. They recommended mechanistic explanation that aldehyde stimulate the activity at greater rate than acid when apo-10′-lycopenal (10′-al), formed from single cleavage of lycopene at the 9′,10′ double bond, was compared to apo-10′-lycopenoic acid. Secondly, there is direct correlation for activity between number of methyl and aldehyde group on both sides of molecule. Linnewiel et al. also studied reactivity of carotenoid aldehyde derivatives through the combined effect of diapocarotene-dials with chain length of 8–12 carbon atoms. The results revealed the correlation between methyl group and terminal aldehyde. Methyl group at gamma or delta position enhances the activity of molecule in cells (LNCap and MCF-7). Further the effect of backbone chain was determined by comparing the activity of derivatives with different numbers of carbon atoms. From which 12 carbon chain compound showed more potency as compared to 16 carbon chain compound displaying that the main chain of molecule containing 12 carbon is optimal for inducing EpRE/ARE transcription system. These results altogether could display structural improvement positions for curing effect of carotenoid (Table 1) [38]. Similar to lycopene, crocetin is also the oxygenated form of carotenoids found in saffron and having anti-tumor activity. Their mechanism includes inhibition of nucleic acid synthesis, growth factor signaling pathway and induction of anti-oxidative system and apoptosis [39].

## 8 Ginger

Use of ginger for medical treatment is popular practice in eastern culture. It is underground rhizome with striated texture. Researchers believe that ginger affect by two methods either through apoptosis or autophagy and evidences associates it with the cause to reduce colorectal and ovarian cancer. Moreover ginger extract helps in reducing the NF-kB expression [40]. In study performed by Dugasani et al. about comparison of antioxidant and anti-inflammatory effect between gingerols and their derivatives to determine the evaluation of scavenging of 1,1-diphenyl-2-picyrlhydrazyl (DPPH), superoxide and hydroxyl radicals, inhibition of *N*-formyl-methionyl-leucyl-phenylalanine induced reactive oxygen species (ROS) construction in human polymorphonuclear neutrophils, inhibition of lipopolysaccharide induced nitrite and prostaglandin E creation in RAW264.7 cells, through SAR studies demonstrated that α, β unsaturated ketone moiety could be the reason for anti-inflammatory and anti-oxidant activity. However the better potency of 10-gingerol instead of parent structure, 8-gingerol, 6-shogaol and 6-gingerol shows the significant role of chain length (Table 1) [41]. Also reverse docking studies performed on [6]-gingerol with leukotriene A_4_ hydrolase (LTA_4_H) as target for cancer therapy confirmed that this compound has binding similarities like bestatin (the aminopeptide inhibitor) which binds to Glu271 residue of LTA_4_H. They supported their prediction by testing and confirming [6]-gingerol inhibitory effect on colorectal cancer cells [42].

## 9 Pau d'arco

Pau d'arco, the inner bark or heartwood of a tropical member of the bignonia family has been studied for its anti-inflammatory, anti-cancer, antibacterial, antifungal, and immuno-stimulant activities [43]. Lapachol as active ingredient in this bark was used to perform SAR studies. Investigations done to synthesize 5-hydroxylapachol having anti-proliferative activity through prenylation of 2-hydroxyjuglone uncovers the structure activity relation of lapachol derivatives. Potency of molecule to suppress tumor cell line depends on the presence and position of hydroxyl group attached to the aromatic ring. This factor was confirmed after comparison between lapachol and its derivatives on human solid tumor cells. A contrast study between hydroxyl groups at carbon position 4, 5, 8 for anti-proliferative activity and fact that presence of phenolic hydroxyl group at C-5 enhances the activity prove the above result. Furthermore at one side the conversion of free hydroxyl groups in the quinone ring to methyl ethers results in improvement in molecule's action while on the other side poorly activated derivatives were produced when prenyl side chain was removed. In addition a decrease in activity was due to the replacement of the exocyclic double bond by an oxygenated function (Table 1). So, conclusion proposes that both size and relative position of side chain are essential for generation of a modified compound with better activity [44].

## 10 Green tea

Polyphenols present in Green tea not only supresses the cancer cell growth but also kill them after distinguishing tumor cells from normal cells. Health filled characteristics have encouraged researchers to work with Green tea compound EGCG having anti-tumor and anti-angeogenic abilities. Scientists say that number of proteins required for tumor suppression are degraded by proteasome [45]. Many investigation studies regarding ECGC suggests that although A-ring with gallate ester/amide bond are essential for proteasome inhibition, the number of hydroxyl groups associated with A, B or G ring are also important for enhancing antitumor activity. More facts declares that hydroxyl groups attached to the C-3 and C-4 position of G-ring also arises the activity level of cytotoxicity (Table 1) [46], [47], [48].

## 11 Vegetable

Consuming vegetables no doubt develop the immune system and other essential components of body to fight against various diseases including cancer growth promoting factors. In order to interpret SAR studies on sulforaphane (active ingredient in vegetables) for high inducer properties racemic sulforaphane and its derivatives different in position and number of oxidation state of sulfur and methylene group were tested on murine hepatoma cells. Additive effect of inducing glutathione transferase and quinone reductase in certain mouse tissues was seen with sulforaphane and its sulfide derivatives. Synthetic and isolated sulforaphane gave approximate similar CD values showing that chirality of sulfoxide does not affect its inducer values whereas other factors like oxidation of the side chain sulfide to sulfoxide or sulfone and compounds with 4 or 5 methylene groups in the bridge linking CH_3_S— and —N=C=S enhances the potency level as compared to three methylene group [49].

## 12 Red clover

Red clover is a fodder crop with nitrogen fixing properties. Besides this it is also taken to lab for having anti-cancer effect. Genistein has been regarded as the anti-cancer compound in red clover. Numerous studies on genistein have shown to affect many biochemical targets like Protein tyrosine kinases, topoisomerase II, estrogen receptor, ABC transporters, enzymes involved in phosphatidylinositol turnover etc. which are basically involved in cancer progression pathway. Zava and Duwe investigated estrogenic effect of genistein on MCF-7 cells and suggested that 4′-hydroxy position on C-ring and 7′-hydroxy position on A-ring could be primary factors responsible for estrogenic activity. As evidence it was seen that alteration made either as methylation at 4′-hydroxy group or changing its conformation by shifting of phenolic B ring from 3- to 2- position bound on pyran ring, resulted in drastic decrease in inhibitory activity. Indeed 5- hyroxyl and 4- ketonic oxygen groups were favorable too (Table 1) [50].

Moreover in case of genistein as PTK inhibitor Ogawara et al. compared structure activity relationship against EGF-receptor with certain isoflavonoids. They analyzed that removal of hydroxyl group at position 5 is essential for proper functioning of the compound. In addition genistein has also shown a capability among (iso) flavonoids to inhibit topoisomerase II [51].

## 13 Marine products

Marine products have also participated in healing process. Researchers have been probing deep under the ocean to find a solution to various diseases. Some are found with anticancer activity while others can reverse back multidrug resistance in cancer cells. Lamellarins inhibit the *P*-glycoprotein whose mechanism includes transporting anticancer agents out of cancer cells and thus making them multidrug resistance [52]. SAR studies concluded many positive and negative factors responsible for anti-tumor activity of lamellarins. Firstly, positive factors includes importance of double bond between C5=C6 position as an essential factor for anti-topoisomerase-I activity and —OH group at C-8, C-14, C-20 position. Hydroxyl group at R1, R2 and R4 could activate the cytotoxicity activity by DNA topoisomerase I inhibition or kinase activity. Secondly discussing the negative factors, hydroxyl attachment at R3, *O*-methylation at R6, replacing R1, R3, R6 with *O*-isopropyl, saturation of double bond between C-5 and C-6, methoxy group at C-13, C-12 and acetylation with various carboxylic acids may leads to massive decline in cytotoxicity level of compound. Furthermore an SAR investigation led by University of Queensland, Australia to evaluate cytotoxity, *P*-glycoprotein inhibition and multidrug resistance reversal activity, 30 lamellarines and their derivatives identified through 2D spectroscopy were utilized and thus reporting correlation between *P*-glycoprotein inhibition and degree of methylation of lamellarin hydroxyl groups. Although in human colon cancer cells highly methylated compounds were capable of reversing multidrug resistance yet less methylated compounds showed enhanced cytotoxic activity (Table 1) [53], [54], [55].

## 14 Tripterygium wilfordii

From many years *Tripterygium wilfordii* has been used as immunosuppressant. Bioactive components with high anti-tumor activity are celastrol and triterpene. Celastrol is also designated as proteasome inhibitor [56]. In case of celastrol studies performed against topoisomerase II indicates that C-1 to C-10 and double bond between C7=C8 are essential while any modifications made at C-6, C-21, C-22, C-23 position decreases the activity of apoptosis shock response and inhibit Hsp90 and most of the time variations made at carboxylic acid by amide group functionality improved potency of compound [57].

In case of triterpene, phosphatidylinositol specific phospholipase C as target which leads to termination of DNA synthesis and cell growth. Lee et al. presented their study which deals with systematic structure activity relationship of triterpene ester against PLCγ1. Derivatives as acetate, methyl ester and reduced form at 2′double bond produced lower inhibitory effect when compared to parent compound. Results suggested the presence of 3-OH, 7′-OH, 28 COOH, 2′ double bond and esterification of triterpene as important features for PLCγ1 inhibitor. Moreover 3-OH, 27 esterification, 28-COOH, 2′double bond and *p*-coumaroyloxy at position 27 may be important for improved inhibitory action (Table 1) [58], [59].

## 15 Coffee

Most consumable beverage in world is coffee. The major phenolic compound in coffee is chlorogenic acid which is an ester of caffeic acid and quinic acid and makes it capable to absorb free oxygen radicals [60]. SAR study has not been performed on this compound yet.

## 16 Grape seeds

Studies performed on grape seed extract states that it brings destruction in leukemic cells. Gallic acid is one of the cancer cells suppressing agent. A study performed on anti-proliferative and cytotoxic activity on HeLa cells presented the structural data on methyl gallate, propyl gallate, octyl gallate and few other polyphenol derivatives by theoretical (ab initio) approach. For improved cytotoxic action of compound major factors includes number of hydroxyl substituents on the ring and the side-chain length between aromatic ring and terminal carboxylate group (Table 1). The study concluded that a slight structural change in derivatives have lead to improved biological activity. For instance di- and trihydroxylated propyl esters produced distinct results than parent methyl and octyl analogs when tested on cell line. Due to effect of variation in length of the alkyl chain long alkyl chains demonstrated an enhancement in activity while short alkyl chain showed low antiproliferative activity. The study was emphasized on properties like size, degree of ring hydroxyl substitution and length of the alkyl chain. In addition, increase in chain length increased the lipophilicity of compound which is also considered as an important factor for drug designing [61], [62], [63].

## 17 Red grapes

Resveratrol (3,4′,5-trihydroxystilbene) an anticancer agent from red grapes prevents cancer by inhibiting cyclooxygenase enzymes and angiogenesis with modulation of drug metabolizing enzymes. Anti-oxidation, alterations in cell cycle and apoptotic machinery simultaneously contribute in a process [64]. Compound with respect to the growth of PC-3 and LNCaP human prostate cancer cells have improved activity for anti-progressive activity. SAR studies suggest the significant presence of two methyl-oxyl groups at position 3 and 5, N at 4′ position and N=C double bond in the connecting chain as four analogs verified potent growth inhibitory activity (IC50 0.01–0.04) in LNCaP cells [65]. Also N replaced by a C— methoxy group decreases the activity while replacement of —OH group to methoxy group increases the activity of compound. *Cis*-form showed higher cytotoxicity when compared among its methoxylated analogs. A study performed to confirm the nature of series of methoxylated analogs of resveratrol revealed that substitution of hydroxyl group with methoxy group in resveratrol produced potential results in anti-tumor studies. In addition the formed derivatives with substitution of group showed better potency in *cis-*form when compared to *trans-*isomers. In case of hydroxylated resveratrol in *trans*-conformation hydroxyl group at position 4- and 4′ act as backbone of compound for anti-tumor effect [66].

## 18 Maitake mushrooms

An active compound in maitake mushrooms is irofulven (hydroxymethylacylfulvene). It mainly promotes the immune system functioning and efficiency of related cells like natural killer cells, cytotoxic T-cells, IL-1, IL-2, lymphokines etc. [67]. Its unique property is to act as selective inducer of apoptosis in human cancer cell lines. Facts disclose that active compound in mushrooms usually work by boosting the immune system. Irofulven stimulates the activity of interleukin-2 mediated lymphokine-activated killer cells and NK cells and instead of attacking directly on cancer cells, immune system is activated. Although in 2001 Food and Drug Administration (FDA) approved irofulven as “fast track” in gemcitabine-refractory pancreatic cancer cases but unfortunately irofulven showed side effects of retinal damage and visual disturbance in Phase II clinical trials due to which investigation on this compound was seized [68]. So, to improve the potency of compound and decrease its side-effect its structurally modified analogs were tested.

Studies performed with analogs recommend that efficiency of Irofulven as anti-tumor agent solely not only depends on free allylic hydroxyl group but also on analog longer chain substituents, followed by hydroxymethyl group (Table 1). It also shows electrophiles, bioreductive activation, cyclopropane ring opening, alkylation of protein and DNA as major factors for biological activity [69]. McMorris et al. investigated on illudin S and M analogs to find compounds selective for normal and infected cells. The key features concluded for anti-proliferative activity were α,β-unsaturated ketones and cyclopropylmethyl carbinol. They also publicized that hydroxymethyl group and long chain substitutions could possibly produce better features for curing tumor cells [70].

## 19 Conclusion

Though many natural components with anti-cancerous characteristics are present and some of them are under clinical trials but still the interest in this part of research is not enough may be due to the reasons of considering natural products less liable to patent-ship. Exploring land and ocean can provide us with the solution to our medical problems if attention is paid toward them. Use of edible and non-edible plants constituents with computational tools to design drugs could enlighten the path toward cancer free approach.
